# Ocular Involvement in Coronavirus Disease 2019: Up-to-Date Information on Its Manifestation, Testing, Transmission, and Prevention

**DOI:** 10.3389/fmed.2020.569126

**Published:** 2020-11-30

**Authors:** Ziyan Chen, Gang Yuan, Fang Duan, Kaili Wu

**Affiliations:** ^1^State Key Laboratory of Ophthalmology, Zhongshan Ophthalmic Center, Sun Yat-sen University, Guangzhou, China; ^2^Department of Geriatrics, The First Affiliated Hospital, Sun Yat-sen University, Guangzhou, China

**Keywords:** COVID-19, SARS-CoV-2, ocular surface, manifestation, testing, transmission, prevention, conjunctivitis

## Abstract

The coronavirus disease 2019 (COVID-19) pandemic, caused by the novel severe acute respiratory syndrome coronavirus 2 (SARS-CoV-2), is still underway. An understanding of the virus's mode of transmission and infection is required for its effective containment. Besides the respiratory and digestive tracts, the ocular surface presents an additional mucosal surface that is exposed to infectious droplets and direct/indirect contact. The relationship between SARS-CoV-2 infection and the eye remains controversial. This review examines up-to-date information on ocular manifestation, laboratory testing, transmission, and prevention of COVID-19. Based on clinical observations, the risk of conjunctivitis in COVID-19 is low. Despite the low incidence, positive SARS-CoV-2 results in eye specimens suggest that the ocular surface may harbor SARS-CoV-2, which may increase the infection and transmission risk. We conclude that the ocular surface remains a potential transmission route for the virus that should not be ignored. In addition, the intraocular findings have also been described in COVID-19. The measures for eye and face protection should be widely adopted to stem the tide of the pandemic.

## Introduction

Coronavirus disease 2019 (COVID-19) is caused by the novel severe acute respiratory syndrome coronavirus 2 (SARS-CoV-2) and has been recognized by the World Health Organization as a global pandemic. COVID-19 was first reported in Wuhan, China in December 2019 (1). As of May 31, 2020, COVID-19 has been confirmed to have affected more than 5,000,000 people with more than 300,000 deaths spanning most countries in the world (2). Coronaviruses have attracted worldwide attention since the outbreak of severe acute respiratory syndrome (SARS) in 2003, caused by the severe acute respiratory syndrome coronavirus (SARS-CoV) (3), and the Middle Eastern respiratory syndrome (MERS) in 2012, caused by the Middle Eastern respiratory syndrome coronavirus (MERS-CoV) (4). The ongoing COVID-19 global pandemic is a vivid reminder of the continuous evolution of microbes. SARS-CoV-2 shares 79.6% sequence identity with SARS-CoV (5). These two viruses bind to the same human cell receptor, namely, angiotensin-converting enzyme 2 (ACE2). Although these viruses are genetically related, SARS-CoV-2 is much more communicable than SARS-CoV (1). Human-to-human transmission of SARS-CoV-2 occurs mainly through respiratory droplets and direct/indirect contact via the mucous membranes. The ocular surface presents an additional mucosal surface that is exposed to infectious droplets and hand contact. Concerns have been raised over whether SARS-CoV-2 could be transmitted through ocular exposure or lead to ocular complications, following reports of a Chinese expert on COVID-19 who was not wearing eye protection during an inspection in Wuhan, and subsequently developed redness of the eyes several days before he was diagnosed with SARS-CoV-2 infection (6). In previous studies, several patients with COVID-19 were observed to exhibit ocular abnormalities (7–10). With the ongoing outbreak, mounting evidence suggests a relationship between SARS-CoV-2 infection and ocular involvement; however, it remains controversial. To analyze the evidence, we performed a review of the published literature and preprints in the following databases: PubMed, World of Science, ScienceDirect, the Cochrane Library, medRxiv, and bioRxiv. We searched for literature published from January 1, 2002 to May 31, 2020, to present up-to-date information on ocular involvement in COVID-19.

## Ocular Manifestation in COVID-19 Patients

Seven kinds of human coronaviruses have been identified. Four of these, HCoV-229E, HCoV-NL63, HCoV-OC43, and HCoV-HKU1, generally cause only mild respiratory tract infections in humans (11, 12). The three most pathogenic human coronaviruses, SARS-CoV-2, SARS-CoV, and MERS-CoV more commonly cause life-threatening pneumonia and even death. Human coronaviruses and their association with ocular diseases have been discussed before. HCoV-NL63 was first isolated from the respiratory specimen of a 7-month-old child with bronchiolitis and conjunctivitis (11). In one study, 3/18 HCoV-NL63 patients (17%) <20 years of age had conjunctivitis (13). Loon et al. (14) detected SARS-CoV in the tears of 3/8 probable SARS patients (38%) in the early course of the disease (within 9 days of onset). However, in a case series reported by Chan et al. (15), neither tears nor conjunctival scraping samples were positive for SARS-CoV in any of the 17 patients confirmed to have SARS, and no virus-associated ocular complications were detected in these confirmed cases. Ocular involvement has not been described with human MERS-CoV infection.

Since the beginning of the current outbreak, a growing amount of clinical reports of ocular manifestations in COVID-19 patients have been published (Table 1), more so than in SARS patients. The incidence of ocular manifestations in COVID-19 is varied and generally low (Table 1). The ocular manifestations described most commonly in COVID-19 patients, apart from discomforts, include unilateral or bilateral conjunctival congestion, hyperemia, chemosis, increased secretions, watery discharges, epiphora, or conjunctival follicles with (16, 18, 19, 21–25, 28, 31, 32) or without (7–10, 17, 20, 27, 30) a diagnosis of conjunctivitis by an ophthalmologist. Subconjunctival hemorrhage (25), pseudomembranes (25, 31), and impaired vision (22) were also reported, although less often. Ocular manifestation can present as an initial symptom (7, 8, 10, 19, 22, 24, 30–32), or an isolated symptom (28), in COVID-19.

**Table 1:** A summary of clinical ocular involvement in COVID-19 and SARS cases.

**Author**	**Study type**	**Region**	**No. of patients**	**No. of patients with ocular manifestations (%)**	**Clinical type (no.)**	**Ocular manifestations**	**Ocular examination methods**	**Ocular manifestations as initial symptoms**	**Duration (days)/onset date (day)**	**Treatment for ocular manifestations**	**Collection methods/virus assays**	**No. of patients with positive virus assay in eye specimens (%)**	**Sampling interval^#^ (days)/sampling date for positive virus assay^$^ (day)**
**COVID-19**													
**Symptoms with ocular virus testing**													
Xia et al. (16)	Prospective case series	Zhejiang China	30	1 (3.3%)	M	Viral conjunctivitis with conjunctival congestion and aqueous secretion	NR	NR	NR	NR	Cj swab/RT-qPCR	1 (3.3%)	7.33 ± 3.82 days/Day 3, 5
Seah et al. (17)	Prospective case series	Singapore	17	1 (5.9%)	NR	Conjunctival injection and chemosis	NR	0	NR	NR	Schirmer's strip tear collection/RT-qPCR/virus culture	0	3 ~ 20 days
Zhang et al. (18)	Cross-sectional study	Wuhan China	72	2 (2.8%)	1 M • 1 NR	Conjunctivitis	Slit-lamp	0	4 days/Day 3 ~ 7	Ganciclovir eye drops	Cj swab/RT-qPCR	1 (1.4%)	18.15 ± 7.57 days
Wu et al. (7)	Retrospective caseseries	Hubei China	38	12 (31.6%)	4 M • 8 S	Conjunctival hyperemia, chemosis, epiphora, and increased secretions	Bedside flashlight	1	NR	NR	Cj swab/RT-qPCR	2 (5.3%)	NR
Ye et al. (19)	Retrospective case series	Wuhan China	30	3 (10.0%)	NR	Conjunctivitis	Slit-lamp/ Fundus	1	7 ~ 10 days	Ganciclovir eye drops	Cj swab/RT-qPCR	2/27 (7.4%)^*^	NR
Zhou et al. (20)	Retrospective case series	Wuhan China	121	8 (6.6%)	7 S • 1 M	Itching, redness, tearing, discharge, and foreign body sensation	Penlight	NR	15.0 ± 8.8 days	NR	Cj swab/RT-qPCR	3 (2.5%)	NR
Chen et al. (21)	Case report	Shenzhen China	1	1	NR	Acute follicular conjunctivitis/unremarkable fundus	Slit-lamp/Fundus/OCT	0	Day 13	Ribavirin eye drops	Cj swab/RT-qPCR	1	Day 14, 17
Cheema et al. (22)	Case report	North America	1	1	M	Unilateral keratoconjunctivitis/unremarkable fundus	Slit-lamp/ Fundus	1	Day 1	Oral valacyclovir, moxifloxacin eye drops	Cj swab/RT-qPCR	1	Day 3
Colavita et al. (23)	Case report	Italy	1	1	NR	Conjunctivitis	NR	0	17 days	NR	Ocular swab/RT-qPCR/virus culture	1	Day 3, 9, 13, 16, 21
Khavandi et al. (24)	Case report	Iran	1	1	NR	Mucoid discharge and follicular conjunctivitis	Slit-lamp	1	Day 1	NR	Cj swab/RT-qPCR	1	NR
Navel et al. (25)	Case report	France	1	1	S	Pseudomembranous andhemorrhagic conjunctivitis/no vitreous inflammation and retinal abnormalities	Slit-lamp/ Fundus	0	Day 17	Azithromycin eye drops, low doses of dexamethasone	Cj scraping & swab/qPCR	0	NR
Hu et al. (26)	Case report	Xian China	1	1	M	None	NR	0	N/A	N/A	Cj swab/RT-qPCR/NGS	1	Positive for two weeks
**Symptoms without ocular virus testing**													
Guan et al. (9)	Cohort study	China	1099	9 (0.8%)	4 S • 5 M	Conjunctival congestion	NR	NR	NR	NR	N/A	N/A	N/A
Abrishami et al. (27)	Cross-sectional study	Iran	142	41 (28.9%)	31 S • 10 M	Conjunctival hyperemia	Slit-lamp/ Fundus	0	NR	NR	N/A	N/A	N/A
Chen et al. (10)	Cross-sectional study	Wuhan China	534	25 (4.7%)	•15 S • 10 M	Conjunctival congestion	Telephone/face-to-face survey	3	4.9 ± 2.6 days	Ganciclovir, tobramycin, ofloxacin eye drops, artificial tears	N/A	N/A	N/A
Hong et al. (8)	Retrospective case series	Zhejiang China	56	15 (26.7%)	9 S • 6 M	Sore eyes, itching, foreign body sensation, tearing, redness, dry eyes, eye secretions, and floaters	Questionnaire	6	NR	NR	N/A	N/A	N/A
Scalinci and Battagliola (28)	Retrospective case series	Italy	5	5	M	Isolated conjunctivitis	Slit-lamp	5	NR	Moxifloxacin eye drops	N/A	N/A	N/A
Marinho et al. (29)	Retrospective case series	Brazil	12	12	M	Hyper-reflective lesions at the level of ganglion cell and inner plexiform layers, subtle cotton wool spots, and microhemorrhages	Fundus/OCT	0	NR	NR	N/A	N/A	N/A
Daruich et al. (30)	Case report	Argentina	1	1	S	Unilateral eyelid edema and moderate conjunctival hyperemia	Telemedicine	1	Day 1	NR	N/A	N/A	N/A
Salducci and La Torre (31)	Case report	Diamond Princess ship	1	1	M	Viral conjunctivitis with transparent serous secretions, conjunctival chemosis, and pseudomembranes	N/A	1	7 days/Day 1	Ganciclovir gel, artificial tears	N/A	N/A	N/A
Wu et al. (32)	Case report	Hubei China	1	1	M	Unilateral conjunctivitis and eyelid dermatitis	Flashlight	1	5 days/Day 5	NR	N/A	N/A	N/A
**SARS**													
Chan et al. (15)	Case series	Hong Kong	20	0	NR	None	NR	N/A	N/A	N/A	Cj swab/RT-qPCR	0	NR
Loon et al. (14)	Case series	Singapore	36	NR	NR	NR	NR	NR	NR	NR	Cj scraping & swab/RT-qPCR	3	Day 3 (case 1), Day 9 (case 2), Day 4 (case 3)
Tong et al. (33)	Case series	Hong Kong	4	NR	NR	NR	NR	NR	NR	NR	CURTI/qPCR	2/4 CURTI specimens	NR
Yuen et al. (34)	Prospective case series	Hong Kong	45	2 (4.4%)	NR	Mildly higher IOP (corticosteroid-related complication)	Slit-lamp/Fundus	NR	NR	NR	N/A	N/A	N/A

*COVID-19, Coronavirus disease 2019; SARS, Severe acute respiratory syndrome; S, severe/critical case; M, mild/moderate case; NR, not recorded; N/A, not applicable; Cj, conjunctival; RT-qPCR, reverse transcriptase quantitative polymerase chain reaction; NGS, next-generation sequencing; OCT, optical coherence tomography; CURTI, conjunctiva-upper respiratory tract irrigation*.

# *An interval from COVID-19 onset to the conjunctival sampling*.

$ *A sampling date after COVID-19 onset for positive result of virus assay*.

* *The data of symptoms from 30 patients and the data of virus testing from 27 patients were from two different isolation wards in this study*.

Although the incidence is low, evidences show that SARS-CoV-2 can infect the ocular surface to cause conjunctivitis. Xia et al. (16) detected viral conjunctivitis in 1/30 COVID-19 patients (3.3%); the patient was a 53-year-old man with moderate COVID-19, conjunctival congestion, and aqueous secretion of the eyes, and SARS-CoV-2 nucleotides were detected in conjunctival swab samples. Zhang et al. (18) discovered that 2/72 COVID-19 patients (2.8%) had conjunctivitis; SARS-CoV-2 nucleotides were detected in the ocular discharges of one of these. The latter, a 29-year-old female nurse, working in an emergency department, presented with persistent bilateral conjunctival congestion, and watery discharges on day 2 with the onset of a moderate fever (day 1). Chen et al. (21) presented a case of a 30-year-old man with COVID-19 who had bilateral acute viral conjunctivitis of moderate hyperemia, a watery discharge, and inferior palpebral conjunctival follicles with a delayed onset (13 days after the onset of systemic disease), when conjunctival swab specimens were positive for SARS-CoV-2. The acute onset of the ocular manifestations with conjunctival hyperemia and watery discharges, and the positive virus testing of ocular samples indicated that it was viral conjunctivitis because of SARS-CoV-2 infection of the ocular surface.

However, not all studies reported the specific ocular manifestations related to SARS-CoV-2 infection (8–10, 27). The varying incidence in different reports may be attributed to the different methodology and varying definitions/criteria of conjunctivitis. Because of the risk to healthcare workers and the critical condition of patients, telemedicine, or penlight examinations were performed in some reported cases instead of slit-lamp microscopy and fundus examinations. Conjunctivitis is known to be a common ophthalmic manifestation with a variety of infectious or non-infectious causes (35). Conjunctivitis in a proportion of COVID-19 patients may have other viral, bacterial, or allergic causes and should be diagnosed differentially, especially in those cases lacking of ocular virus testing (28, 30, 32). The conjunctival congestion and chemosis, which mimic the symptoms of conjunctivitis, exhibited by COVID-19 patients (9, 27) may be related to mechanical ventilation, electrolyte disturbances, and fluid overload, especially in severe cases in intensive care units (ICUs) (36, 37). A study from Iran demonstrated that patients with COVID-19 in ICU were at an increased risk of developing chemosis (27). In addition, prolonged periods of reading during the isolation may be the cause of dry eye in COVID-19 patients (8, 10).

Another concern is that the cornea and intraocular tissues may be affected by COVID-19. Cheema et al. (22) reported a case of a 29-year-old female who was diagnosed with COVID-19 after initial presentation of unilateral keratoconjunctivitis with subepithelial infiltrates and overlying epithelial defects that spread diffusely through the entire cornea; a conjunctival swab sample was positive for SARS-CoV-2. Interestingly, it has been reported that ACE2 is expressed in the retina (38) and aqueous humor (39). Moreover, coronaviruses have been reported to cause retinopathy and optic neuritis in animal models (40). Marinho et al. (29) reported retinal and optical coherence tomography (OCT) changes in patients with COVID-19 and observed hyper-reflective lesions at the level of the ganglion cell and inner plexiform layers, with no visual disturbance or intraocular inflammation in either eye of any of the patients. However, the authors did not test for the SARS-CoV-2 virus in the aqueous humor or vitreous body of those patients. SARS-CoV-2 may target not only the ocular surface but also intraocular tissues; however, more researches are required to determine the scope of its ocular targets. It also suggests the importance of performing full eye examinations in COVID-19 patients.

As for the treatment, there is no proven specific therapy against ocular SARS-CoV-2 infection. Topical antiviral (ganciclovir, valacyclovir, or ribavirin) medicine, antibiotic medicine, corticosteroid eye drops, and artificial tears were applied in some reports (10, 18, 19, 21, 22, 25, 28, 31). However, the therapeutic effect of these medicine was required to be evaluated further. Most ocular manifestations in COVID-19 were mild, and recovered in a relatively short duration, without severe and sight-threatening complications detected (7, 18, 20, 21).

## Laboratory Testing of SARS-COV-2 on the Ocular Surface

During the COVID-19 pandemic, a variety of nucleic acid and serological assays were established for the laboratory-based diagnosis of SARS-CoV-2 (41, 42). Ocular testing for SARS-CoV-2 is most commonly conducted by amplifying virus RNA using reverse transcription quantitative polymerase chain reaction (RT-qPCR) in conjunctival swabs samples or tear fluids. In a few studies, isolating the virus and culture were performed (17, 23). The primer sets used for RT-qPCR of the virus in the respiratory tract and in other specimens, targeting open reading frame 1ab, as well as the nucleocapsid, envelope, spike (S), and RNA-dependent RNA polymerase genes (41, 43), can also be used for ocular specimens. A cycle threshold (*C*_t_) value of 40 or higher is interpreted as negative for SARS-CoV-2 RNA (44, 45). No immunoglobulin or cytokine tests for ocular specimens have been described, as far as we know.

Nucleic acid assays have demonstrated that SARS-CoV-2 can infect the ocular surface and cause conjunctivitis, yet the rate of RT-qPCR tests positive for SARS-CoV-2 RNA in ocular specimens appeared to be low (Table 1). Detection of SARS-CoV-2 in conjunctival or tear specimens may depend on viral load and shedding, as well as timing of sampling during the disease course. The SARS-CoV-2-positive conjunctival swab samples reported by Xia et al. (16) were obtained at the early stages of the disease, on days 3 and 5 after the onset of conjunctivitis. In the case reported by Chen et al. (21), the viral loads in conjunctival specimens collected on days 1 and 2 after conjunctivitis onset (*C*_t_: 31) were much lower than those in nasopharyngeal (*C*_t_: 23.52) and sputum (*C*_t_: 25) specimens. The viral load decreased until it was undetectable on days 5 to 7 after conjunctivitis onset. Hence, we infer that SARS-CoV-2 is prone to exist on the ocular surface during the early stages of conjunctivitis, as verified in a study by Colavita et al. (23). They detected SARS-CoV-2 RNA in ocular swab specimens of a COVID-19 patient with conjunctivitis on days 3, 9, 13, 16, and 21 after symptom onset, even though it was undetectable in nasal swab specimens on day 19. Furthermore, they observed a cytopathic effect 5 days after inoculating Vero E6 cells with the first positive ocular specimen. These findings suggested that the ocular surface of SARS-CoV-2-positive patients may become infectious during the early stages of the disease (23). However, in a study by Seah et al. (17), Schirmer's test strips were used to collect tears on days 3 to 20 after the onset of systemic symptoms, and Vero E6 cells were inoculated with these samples for 4 days. Neither cytopathic effects in Vero E6 cells nor SARS-CoV-2 RNA was detected in 64 tear samples from 17 COVID-19 patients. Low viral loads (below the sensitivity of the tests) or inappropriate timing of sampling may have resulted in false-negative results. On the other hand, very low prevalence (0 ~ 7.4%) of ocular viral findings was reported in six studies with dozens to more than one hundred patients (Table 1). We cannot rule out the false-positive results due to the contamination with hands or testing items. Nevertheless, because SARS-CoV-2 infection is a life-threatening disease, further studies are required to clarify whether and how this virus could be transmitted through the eyes.

Meanwhile, it is noteworthy that SARS-CoV-2 nucleotides have been detected on the ocular surface of patients without ocular symptoms. Zhou et al. (20) discovered positive conjunctival swab samples for 3/121 patients, two of whom did not have ocular symptoms. Ye et al. (19) detected SARS-CoV-2 RNA in conjunctival specimens of 2/27 COVID-19 patients without conjunctivitis. Hu et al. (26) reported a case of a 70-year-old COVID-19 patient with a history of obstruction of the common lacrimal duct in his left eye but did not have conjunctivitis. Nasopharyngeal swab samples were positive for 22 days in this patient, yet ocular swab samples remained positive for 2 weeks after the former became negative. These cases indicate that the ocular surface may harbor SARS-CoV-2, where it may cause a latent and asymptomatic infection without entering the epithelial cells.

## Transmission and Infection of SARS-COV-2 *via* the Eye

As indicated above, the ocular surface has been suggested as a potential target tissue for infection by SARS-CoV-2. However, in a review, Sun et al. (46) considered the ocular surface a less likely route of infection, because of the low prevalence of SARS-CoV-2 detected on the ocular surface and related conjunctivitis in COVID-19. They did, nevertheless, caution that the virus may be transmitted during ophthalmic practice. Peng et al. (47) stated that detection of SARS-CoV-2 RNA in tears and conjunctival secretions of COVID-19 patients with conjunctivitis may be a coincidence, rather than indicating SARS-CoV-2 infection of the conjunctiva as the cause of conjunctivitis.

The S protein of human coronaviruses is responsible for binding to host receptors and entry into host cells, by fusing the viral membrane with that of the host cell, a critical step for viral transmission and infection (48). The SARS-CoV-2 S protein has a receptor-binding domain with amino acids (49) that enhance binding to ACE2, and it has a higher affinity for ACE2 than the SARS-CoV S protein has (50). The S protein of SARS-CoV-2 binds to ACE2, after which it is cleaved and activated by transmembrane protease, serine 2 (TMPRSS2) (51). Moreover, it has been reported that the viral shedding pattern of SARS-CoV-2 appears different from that of SARS-CoV (45, 52). Compared with those of SARS-CoV, higher nasopharyngeal viral loads of SARS-CoV-2 were detected soon after the onset of symptoms (45, 52).

The distribution and expression of ACE2 in the human body may indicate potential infection routes of SARS-CoV-2. ACE2 is widely expressed, including in the lungs, oral and nasal mucosa, nasopharynx, ileum, colon, liver, and kidneys (53, 54). *ACE2* and *TMPRSS2* are also known to be expressed in the epithelium of the ocular surface. By single-cell RNA-sequencing of healthy donors, Sungnak et al. (54) determined that, in the eye, *ACE2* was expressed in the conjunctival, corneal, and limbal epithelial cells; of these, *ACE2* was co-expressed with *TMPRSS2* in the conjunctival epithelial cells. However, it appeared to be inappropriate for the classification of cell types on the ocular surface in this study (54). The expression levels of these two genes were lower in the conjunctiva than in the lungs (54), which might indicate a lower risk for SARS-CoV-2 transmission through the ocular surface than via the respiratory tract. Based on immunostaining data of mice eyes and transcriptomic data of human conjunctival tissue, Zhang et al. (55) discovered that Ace2 and Tmprss2 were expressed in a similar pattern, with high levels in the mouse corneal epithelium, conjunctival epithelium, and lacrimal gland serous cells. The expression level of human *ACE2* and *TMPRSS2* was statistically significantly higher in the conjunctiva than in the cornea, which was consistent with the results in mice. In a study by Ma et al. (56), consistent expression of *ACE2* was detected in human conjunctival and pterygium cell lines from some pterygium patients. The authors also discovered high expression levels of *Ace2* and *Tmprss2* in mouse corneas and suggested that the cornea, rather than conjunctiva, has the highest for SARS-CoV-2 infection. The distribution of *ACE2* on the ocular surface indicates that SARS-CoV-2 may enter epithelial cells of the ocular surface and replicate there, as demonstrated by Hui and colleagues (57).They performed an *ex vivo* study using human conjunctival explant cultures, observing that these were more extensively infected by SARS-CoV-2 than by SARS-CoV, with higher infectious viral titers 48 h post infection. Thus, they provided direct evidence for the potential of the conjunctival epithelium to be a portal of infection for SARS-CoV-2. However, in a study by Lange et al. (58), *ACE2, TMPRSS2*, and other auxiliary mediators were not substantially expressed in healthy or diseased human conjunctival samples at the mRNA and protein level. Thus, the authors suggested that it is unlikely that SARS-CoV-2 infects the conjunctiva via ACE2 and its auxiliary mediators.

There is also anatomical evidence supporting the possibility that the ocular surface is a transmission route of SARS-CoV-2. The ocular surface, which comprises the tear film and the epithelia of the conjunctiva and cornea, is closely linked to the respiratory tract via the nasolacrimal system (59). Blinking spreads, mixes, and distributes tears and generates a pumping effect that draws tears into the lacrimal sac and then to the inferior meatus of the nose (59, 60). The nasolacrimal system therefore forms a route for viruses to spread between the eye and the upper respiratory tract. However, it is unknown in which direction SARS-CoV-2 would spread along this route. Tong et al. (33) exploited this connection, using conjunctiva-upper respiratory tract irrigation to test for the presence of SARS-CoV nucleotides. Patients self-administered one drop of saline to each eye, blinked repeatedly, tilted their head backward, and sniffed to facilitate drainage into the nasopharynx. In two of four confirmed SARS patients, these specimens tested positive, whereas none of the nasopharyngeal swab or stool specimens were positive. Deng et al. (61) performed a single conjunctival inoculation of 1 × 10^6^ 50% tissue culture infectious doses SARS-CoV-2 on two rhesus macaques, and the same was performed via the trachea in another macaque. They detected viral loads in conjunctival swab samples on the first day post inoculation (dpi) in macaques inoculated via the conjunctival route; on the days thereafter, the virus became undetectable in such samples. Viral loads were detected in nasopharyngeal swabs from 1 to 7 dpi in all three animals, while no viral loads were detected in conjunctival swab samples of macaques inoculated via the intratracheal route. Those infected via the conjunctival route had high viral loads in the nasolacrimal system and lower, localized viral loads in the lungs, whereas viral replication was highest in the lungs in those infected via the intratracheal route. These results indicated that rhesus macaques can be infected with SARS-CoV-2 by conjunctival exposure and the systemic condition may be mild. Deng et al. (61) also determined the viral distribution in more detail by euthanizing macaques and performing necropsies at 7 dpi. The results were directly supportive of the hypothesis that SARS-CoV-2 could infect ocular surface epithelial cells and drain into the nasopharyngeal tract via the nasolacrimal system. Although not investigating ocular exposure in isolation, others have studied SARS-CoV (62) and MERS-CoV (63, 64) infection in macaque models, which also indicated that the ocular surface might be a transmission route of human coronaviruses.

## Prevention in Ophthalmology

The rapidly growing number of COVID-19 patients has prompted countries around the world to take measures to control and prevent the transmission of SARS-CoV-2. The ocular surface is a potential route of such transmission through the deposition of respiratory droplets on a surface followed by hand-eye contact, or through aerosolized droplets. Therefore, the potential need for the public and healthcare providers to protect their eyes should not be ignored, especially in areas with high infection rates. Patients with asymptomatic or presymptomatic infections may present to ophthalmologists with initial or isolated conjunctivitis associated with SARS-CoV-2. Therefore, such healthcare providers face an occupational risk during the pandemic. A couple of reports have provided guidance to ophthalmological clinics as precaution to protect patients and healthcare providers (65, 66).

Ophthalmological consultation and care have been altered, including the suspension of elective clinical services and the increased use of telemedicine. All patients and their companions are screened with non-contact thermometers and questionnaires at the entrance to the clinic. Symptoms of upper respiratory tract infections and acute conjunctivitis in patients have also been screened (37, 65, 67). Qiao et al. (68) reported an overall incidence rate of symptomatic COVID-19 infections among eye professionals in 10 hospitals in Wuhan was 2.52%. The authors highly recommended the use of personal protective equipment, a lack of which was one of the risk factors for symptomatic COVID-19 (68). It is important for healthcare workers to use face and eye protection and practice good hand hygiene, in order to protect exposed mucous membranes of the respiratory tract and ocular surfaces (69, 70). Personal protective equipment includes face masks (N95 respirators and surgical masks), goggles, face shields, and gloves (65, 66). To lower the risk of transmission via droplets, protective shields have been installed on slit lamps (67). The use of non-contact tonometry machines has been suspended to prevent possible aerosolization (67). Protective strategies have been recommended for cataract surgery, glaucoma care, corneal transplants, and the management of vitreoretinal diseases (71–74).

## Conclusion

Transmission routes for human coronaviruses are still not completely elucidated. Despite the low prevalence of ocular manifestations compared with respiratory and other systemic disorders, SARS-CoV-2 may be able to infect the ocular surface or intraocular tissues. Further studies should therefore be performed on the interaction between SARS-CoV-2 and the human eye. As the ocular surface may be an additional transmission route, measures should be provided to protect patients' and healthcare workers' eyes from the virus to assist in stemming the tide of the pandemic. Ophthalmologists should take special precaution by wearing eye and face protection, practicing good hand hygiene, and avoiding direct/indirect mucosal contact with patients. Finally, the ocular surface might present a novel treatment route for vaccine delivery.

## Author Contributions

ZC and GY collected the data as well as drafted and revised the manuscript. FD revised the manuscript. KW conceptualized and designed the study as well as reviewed and revised the manuscript. All authors approved the final manuscript as submitted and agreed to be accountable for all aspects of the work.

## Conflict of Interest

The authors declare that the research was conducted in the absence of any commercial or financial relationships that could be construed as a potential conflict of interest.

## Abbreviations

COVID-19: coronavirus disease 2019
SARS-CoV-2: severe acute respiratory syndrome coronavirus 2
ACE2: angiotensin-converting enzyme 2
SARS-CoV: severe acute respiratory syndrome coronavirus
MERS-CoV-2: Middle Eastern respiratory syndrome coronavirus
RT-qPCR: reverse transcription quantitative polymerase chain reaction
C_t_: cycle threshold
S: spike
TMPRSS2: transmembrane protease serine 2.
